# Human trophoblasts are primarily distinguished from somatic cells by differences in the pattern rather than the degree of global CpG methylation

**DOI:** 10.1242/bio.034884

**Published:** 2018-07-19

**Authors:** Teena K. J. B. Gamage, William Schierding, Peter Tsai, Jackie L. Ludgate, Lawrence W. Chamley, Robert J. Weeks, Erin C. Macaulay, Joanna L. James

**Affiliations:** 1 Department of Obstetrics and Gynaecology, The University of Auckland, Auckland 1142, New Zealand; 2 Department of Pathology, Dunedin School of Medicine, University of Otago, Dunedin 9016, New Zealand

**Keywords:** Placenta, Trophoblast, Somatic cells, hESC, Trophectoderm, DNA methylation

## Abstract

The placenta is a fetal exchange organ connecting mother and baby that facilitates fetal growth *in utero*. DNA methylation is thought to impact placental development and function. Global DNA methylation studies using human placental lysates suggest that the placenta is uniquely hypomethylated compared to somatic tissue lysates, and this hypomethylation is thought to be important in conserving the unique placental gene expression patterns required for successful function. In the placental field, methylation has frequently been examined in tissue lysates, which contain mixed cell types that can confound results. To better understand how DNA methylation influences placentation, DNA from isolated first trimester trophoblast populations underwent reduced representation bisulfite sequencing and was compared to publicly available data of blastocyst-derived and somatic cell populations. First, this revealed that, unlike murine blastocysts, human trophectoderm and inner cell mass samples did not have significantly different levels of global methylation. Second, our work suggests that differences in global CpG methylation between trophoblasts and somatic cells are much smaller than previously reported. Rather, our findings suggest that different patterns of CpG methylation may be more important in epigenetically distinguishing the placenta from somatic cell populations, and these patterns of methylation may contribute to successful placental/trophoblast function.

## Abstract (precis)

**Summary:** The placenta may not be as uniquely hypomethylated as previously reported, rather differences in the pattern of CpG methylation are what make it epigenetically distinct.

## INTRODUCTION

During embryonic development, the first cell lineage differentiation event (the separation of the trophectoderm from the inner cell mass) has been associated with the establishment of distinct DNA methylation patterns between these two lineages. Evidence from murine studies shows that the trophectoderm, which will give rise to placental trophoblasts, becomes significantly less methylated than the inner cell mass, which will form the embryo (and ultimately the somatic tissues) (Bianco-Miotto et al., 2016; Schroeder et al., 2015). However, more recent work on human embryos suggests that this difference is not as great in humans as it is in the mouse (Guo et al., 2014; Smith et al., 2014). Nonetheless, these ‘placenta-specific’ patterns of epigenetic modification are thought to influence human placental development and function throughout gestation (Koukoura et al., 2012).

**Fig. 1.**
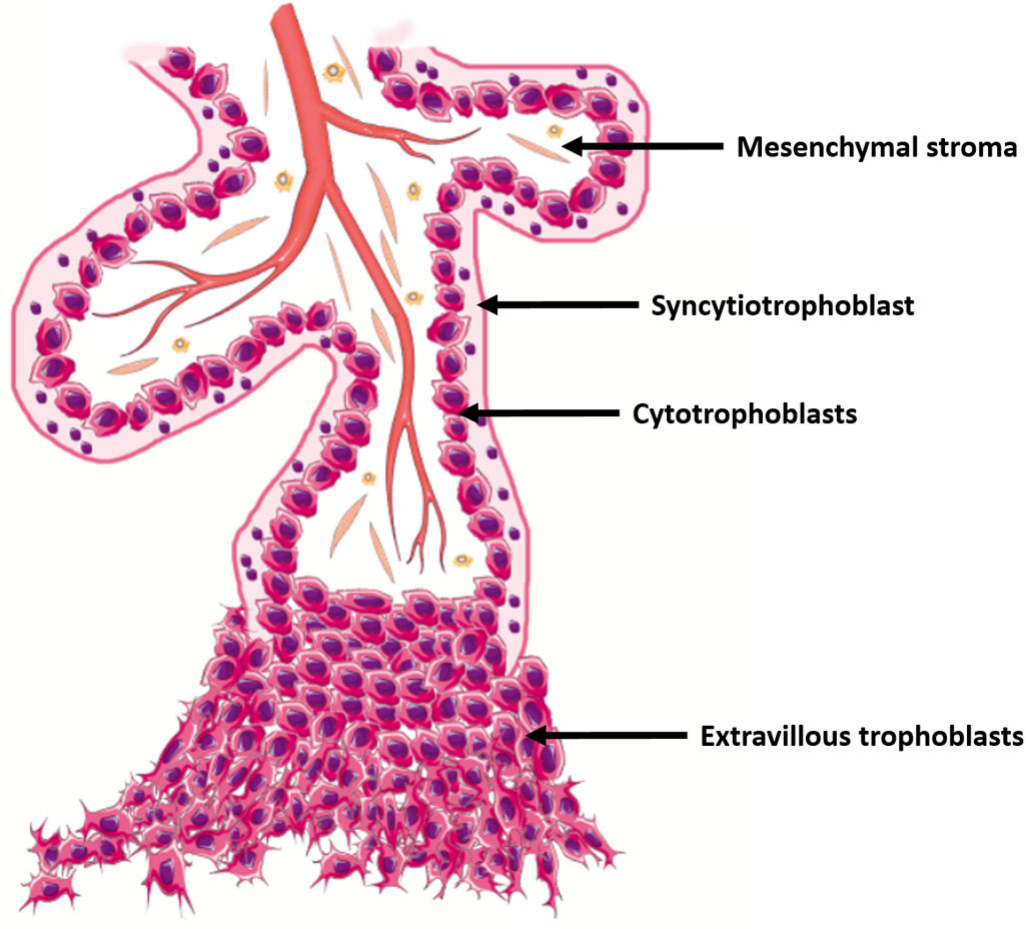
**Schematic diagram of a first trimester placental villus.**

The human placenta has a branching villous structure. Each placental villus has an outer bilayer of trophoblast surrounding an inner core of mesenchymal stroma and fetal blood vessels, thus the placenta itself is comprised of a mix of cells derived from the trophectoderm and the inner cell mass (Boyd and Hamilton, 1970; Luckett, 1978). The trophoblast bilayer comprises a proliferative population called cytotrophoblasts that fuse to form the overlying syncytiotrophoblast layer, which is bathed in maternal blood for most of gestation (Fig. 1) (Boyd and Hamilton, 1970). Cytotrophoblasts also give rise to a second cell population, termed extravillous trophoblasts that invade and transform the uterine spiral arteries to ensure adequate maternal blood flow to the placenta (Fig. 1) (Boyd and Hamilton, 1970; Pijnenborg et al., 1980).

The first key piece of information researchers coming to the field of placental epigenetics learn is that ‘the placenta is a globally hypomethylated organ’, and this hypomethylation is thought to be maintained throughout gestation (Schroeder et al., 2015). Global methylation changes are associated with developmental stages and pathological conditions and can influence cell differentiation (Jackson et al., 2004; Keil and Vezina, 2015). In the placenta, global hypomethylation has been hypothesised to fulfil a uniquely conserved functional role, regulating the gene expression necessary for adequate placental development (Bianco-Miotto et al., 2016; Schroeder et al., 2015). Notably, most research in this field has compared whole-term placental lysates to digested somatic tissues (heart, liver, lungs, spleen, brain, thymus, kidney, whole blood, lymphocytes, neutrophils, and natural killer cells) to show that placental lysates have 14–25% less global DNA methylation than somatic tissues (Chatterjee et al., 2016; Fuke et al., 2004; Novakovic et al., 2010; Schroeder et al., 2013; Tsien et al., 2002). However, as the epigenetic field matures, it is becoming apparent that the cell composition of an organ significantly impacts the degree of methylation reported (Loh et al., 2010; Reinius et al., 2012). Therefore, in order to completely understand how methylation impacts placental function and development, it is essential to look at the individual cell types within the placenta.

While some research has investigated the methylation of single cell populations isolated from the human placenta (Grigoriu et al., 2011), there is limited data comparing individual trophoblast populations to each other, or to isolated somatic cell populations. In this work, we have used reduced representation bisulfite sequencing (RRBS) analysed after the removal of CpG islands (Ficz et al., 2013) to compare the pattern and degree of global CpG methylation of individual trophoblast populations and to cell populations derived from the early blastocyst (trophectoderm, inner cell mass and hESC), and somatic adult tissues. Our findings demonstrate that while individual trophoblast populations of the placenta have low levels of global CpG methylation compared to some somatic cell types, this degree of methylation is by no means as unique to the placenta as has been previously suggested (Chatterjee et al., 2016; Fuke et al., 2004; Novakovic et al., 2010; Schroeder et al., 2013; Tsien et al., 2002). Rather than being distinctly hypomethylated, our findings suggest that the placenta is epigenetically distinct from somatic cells as a result of different patterns of CpG methylation.

## RESULTS AND DISCUSSION

**Fig. 2.**
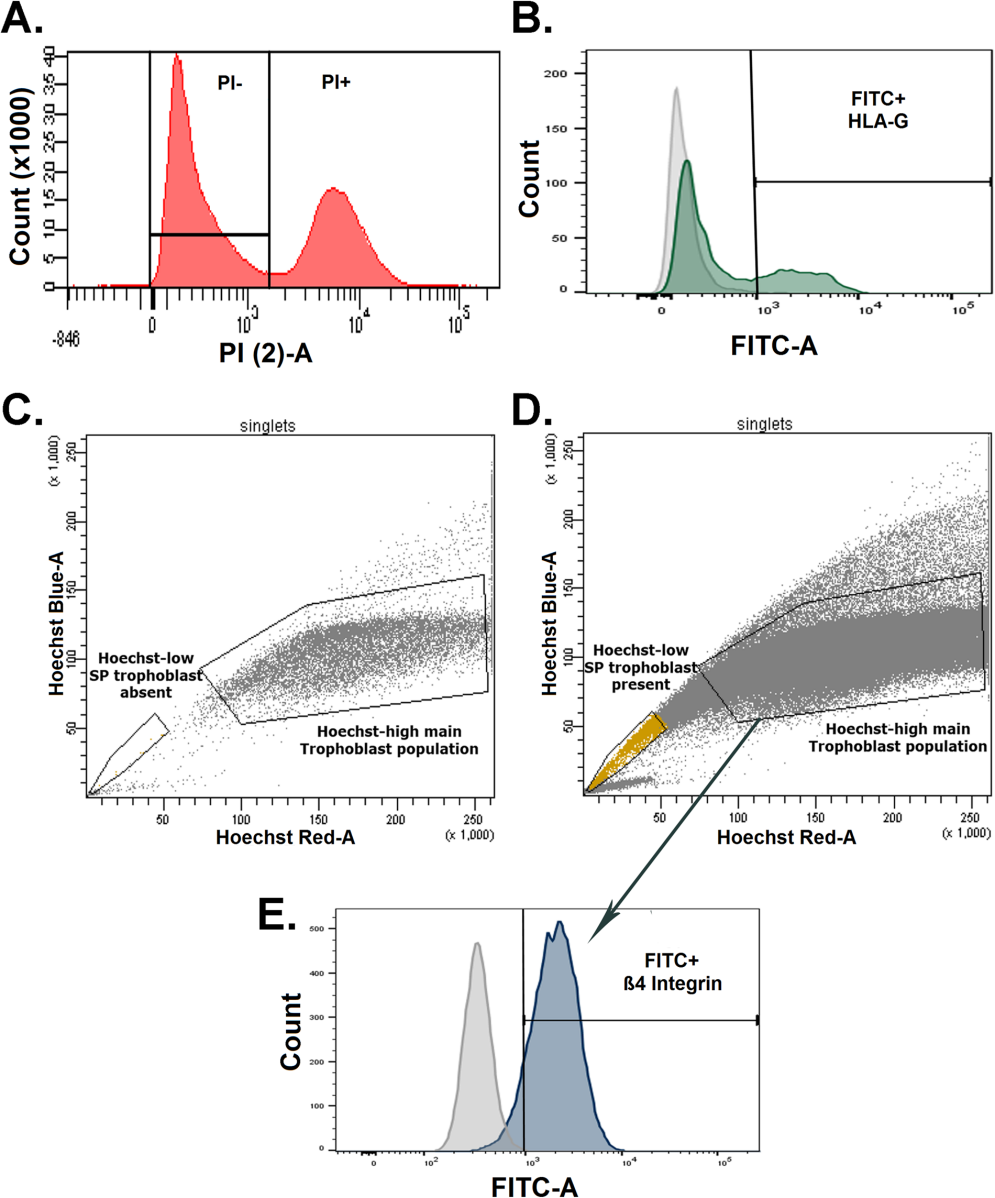
**Isolation of first trimester human trophoblast populations using FACS.** FACs plots showing: (A) the exclusion of non-viable propidium iodide (PI+) and the selection of propidium iodide negative (PI−) cells that progressed through the workflow. (B) Selection of HLA-G positive extravillous trophoblasts. (C) Hoechst 33342 fluorescence intensity of negative control digests treated with fumitremorgin-C and reserpine (inhibitors of Hoechst efflux) showing an absence of cells in the side-population gate. (D) Hoescht 33342 fluorescence intensity of trophoblast digests demonstrating the gating of Hoechst-low side-population trophoblasts. (E) Selection of ß4 positive cytotrophoblasts from the main trophoblast population in D. Cells in B were obtained from the initial villus digest, whereas cells in sorts in C, D and E were obtained from the second overnight digest.

In order to examine the global DNA methylation levels of individual trophoblast populations and compare them to that of other cell types, three distinct primary trophoblast populations – Hoechst side-population trophoblasts [a candidate trophoblast-stem cell population (James et al., 2015)], villous cytotrophoblasts and extravillous trophoblasts – were isolated from first trimester placentae (Fig. 2) and subjected to RRBS. RRBS data for normal hESC, inner cell mass, trophectoderm, first trimester placental lysates, dermal fibroblasts, oesophageal epithelium, renal cortical epithelium, pulmonary epithelium, hepatocytes, astrocytes, B cells, neutrophils and skeletal muscle were sourced from NCBI Geo (Table 1) and all 16 human cell/tissue datasets were analysed using the R package ‘methylKit’ (Akalin et al., 2012).

**Table 1.**
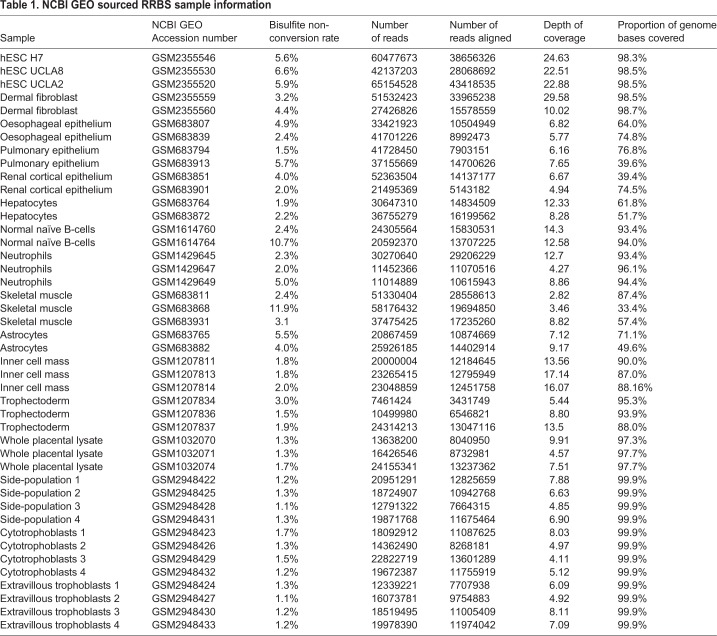
**NCBI GEO sourced RRBS sample information**

**Fig. 3.**
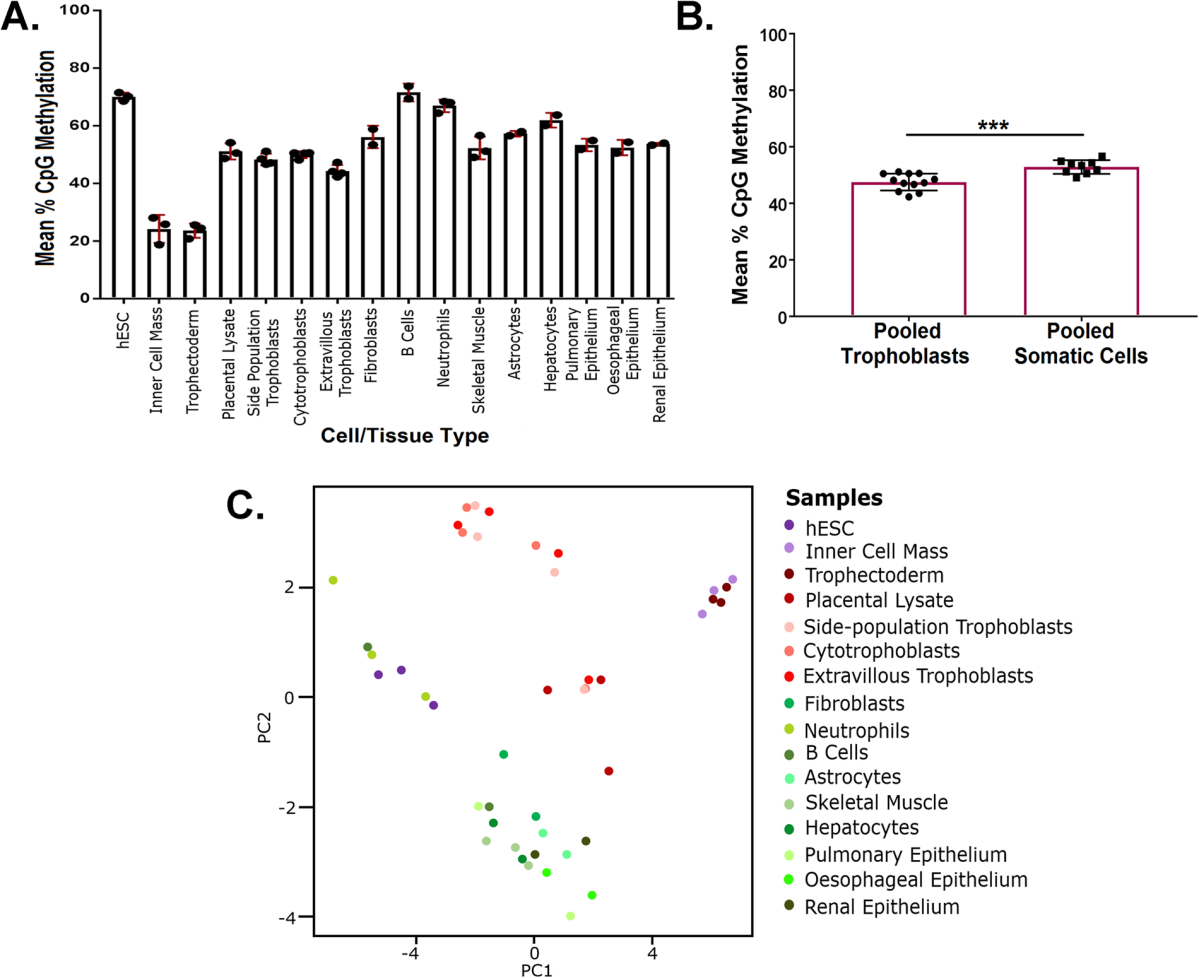
**Percentages and patterns of CpG methylation across human cell and tissue types.** (A) Bar graph showing the mean percentage of methylated CpG sites. There were no significant differences between placental lysates and/or any of the three individual trophoblast populations examined (side-population trophoblasts, cytotrophoblasts or extravillous trophoblasts, *n*=4 of each, *P*>0.24) and all of these populations were significantly more methylated than trophectoderm (*n*=3, *P*<0.0001). All three trophoblast populations were significantly less methylated than neutrophils, B cells and hepatocytes (*P*<0.01). The mean global methylation of side-population trophoblasts, cytotrophoblasts and placental lysates was not significantly different to that of epithelial (pulmonary, oesophageal and renal epithelium) and non-epithelial (skeletal muscle) somatic cell types. Error bars are S.E.M. (B) Bar graph showing the mean percentage of CpG methylation in pooled ‘low methylated’ somatic populations (renal, pulmonary and oesophageal epithelium and skeletal muscle, *n*=9 total) and pooled trophoblast (side-population trophoblasts, cytotrophoblasts and extravillous trophoblasts, *n*=12 total, ***P=0.0003. Error bars are S.E.M. (C) Principal components analysis showing the distribution of cell types using the major (PC1) and minor (PC2) methylation variations present in the RRBS data.

Our analysis reveals that there was no significant difference in the levels of global CpG methylation between the inner cell mass (24.2% methylated, *n*=3) and the trophectoderm (23.6% methylated, *n*=3). This is in contrast to murine blastocysts, where the trophectoderm is reported to be significantly less methylated than the inner cell mass (Schroeder et al., 2015). However, it confirms previous findings from human embryos that showed similar levels of methylation in both the inner cell mass and trophectoderm (Guo et al., 2014; Smith et al., 2014). Interestingly, hESCs, which we originally included in the analysis as a proxy reference for the inner cell mass, were significantly more methylated (70.1% methylated, *n*=3) than cells derived directly from the inner cell mass (*P*<0.0001, Fig. 3A). Furthermore, PCA analysis of global CpG methylation demonstrates that the inner cell mass and trophectoderm cluster together, but both of these cell types separate distinctly from hESC (Fig. 3C). These data highlight the extent of change that hESC derivation or culture may have on these cells and is in line with previous observations (Smith et al., 2014).

There was no significant difference in global CpG methylation between any of the three isolated trophoblast populations (44–50% methylated, *n*=4 isolates of each population, *P*>0.24), nor between any of the trophoblast populations and first trimester placental lysates (51% methylated, *n*=3, *P*>0.09). This is in line with previously published data showing a similar level and pattern of methylation between first trimester cytotrophoblasts and placental villous tissue lysates (Nordor et al., 2017). Recent data employing a novel human trophoblast stem cell population has suggested that these cells are less methylated (33% methylated) than cytotrophoblasts (52.3% methylated) (Okae et al., 2018). However, we did not observe this relationship between side-population trophoblasts (our candidate trophoblast stem cell population) and cytotrophoblasts.

When cell types were analysed individually, the amount of global CpG methylation of placental lysate samples, side-population trophoblasts and cytotrophoblasts was also not significantly different to that of many somatic cell types including pulmonary epithelium (53%), oesophageal epithelium (52%), renal epithelium (54%), and skeletal muscle (52%) (Fig. 3A). However, when all trophoblast samples were pooled and compared with pooled ‘low methylated’ somatic samples (renal, pulmonary and oesophageal epithelium and skeletal muscle), there was a small but significant decrease in global CpG methylation in trophoblasts (47.5%±0.9% s.e.m., *n*=12) compared to the ‘low methylated’ somatic cells (52.8%±0.8%, *n*=9, *P*<0.0003) (Fig. 3B). This 5.3% difference is much less than previous reports (14–25%) obtained from whole tissue lysates, and the biological relevance of such a small difference is unclear (Chatterjee et al., 2016; Ehrlich et al., 1982; Fuke et al., 2004; Gama-Sosa et al., 1983; Schroeder et al., 2015; Tsien et al., 2002). Our analysis also reveals that placental tissue and trophoblasts have a unique pattern of global methylation compared to somatic cells as they cluster distinctly by PCA analysis (Fig. 3C). Taken together, these analyses suggest that the importance of CpG methylation in regulating the highly specialised process of human placentation may not be a result of large differences in the extent of global CpG methylation, but rather may reflect differences in the location of methylated sites within these tissues leading to a distinct pattern of gene expression.

**Fig. 4.**
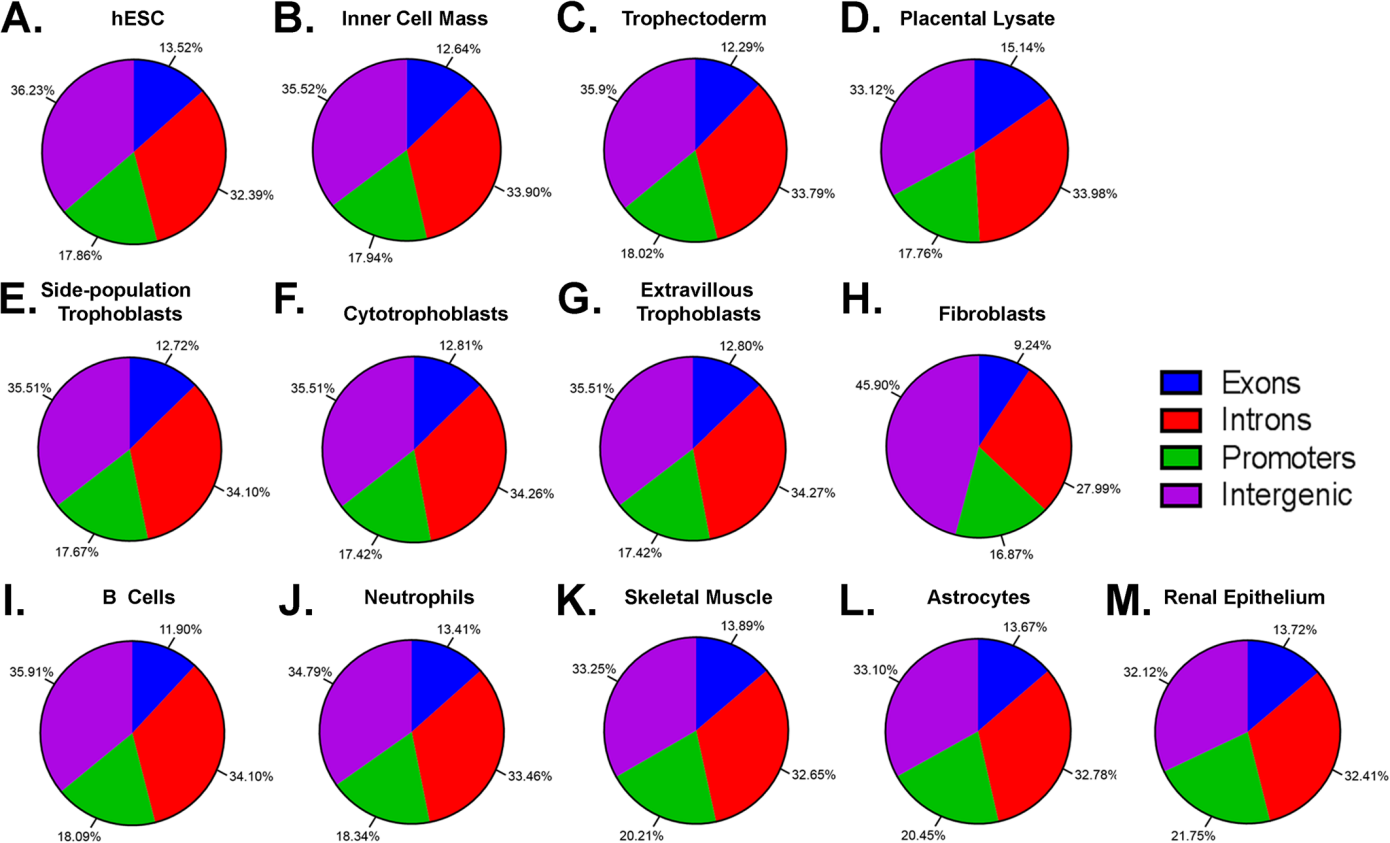
**CpG methylation in promoter, exon, intron and intergenic regions.** Pie charts showing the percentage methylation in promoter, exon intron and intergenic regions in (A) human embryonic stem cells (hESC), (B) inner cell mass, (C) trophectoderm (D) placental lysate, (E) side-population trophoblasts, (F) cytotrophoblasts, (G) extravillous trophoblasts, (H) fibroblasts, (I) B cells, (J) neutrophils, (K) skeletal muscle, (L) astrocytes, (M) renal epithelium.

Whether cell type differences may be attributed to CpG methylation events in various genomic elements was further investigated. The majority of cell types, regardless of their organ of origin, exhibited a very similar distribution of methylation across the genome, with over half of methylation events occurring in introns, intergenic regions and promoters where they can directly influence cell specific gene expression (Fig. 4). Interestingly, in comparison to all other cell types, fibroblasts exhibited significantly more CpG methylation in intergenic regions (46% versus 32–36%, *P*<0.0001) and significantly less CpG methylation in introns (28% versus 32–34%, *P*<0.03). Differences between fetal and adult tissues were also observed, with significantly less promoter CpG methylation (18%) in all fetal tissues (hESC, inner cell mass, whole placental lysate, and all trophoblast populations) compared to skeletal muscle, astrocytes, pulmonary epithelium, renal epithelium and oesophageal epithelium (20–22%, *P*<0.05). These differences in promotor methylation may potentially reflect a greater level of plasticity in fetal cells and tissues in comparison to cells from adult tissues that are more lineage restricted. In this scenario it is possible that increased promoter methylation in cells from adult tissues may result in lineage specific restrictions in gene expression (Koh and Rao, 2013). Less than 16% of CpG methylation events occurred in exons across all cell types. The percentage of CpG methylation events at exons was significantly lower in fibroblasts (9%) compared to trophoblasts, trophectoderm or hESC (11–15%, *P*<0.003). Finally, trophoblast populations were less methylated (13%) in promoter regions than whole placental lysates (15%, *P*≤0.0001) which may be reflective of the mixed tissue type of the whole placenta.

Previous reports have concluded that placental tissue is 14–25% less methylated than somatic tissue, and have largely attributed this to hypomethylation of trophoblasts within the placenta (Chatterjee et al., 2016; Ehrlich et al., 1982; Fuke et al., 2004; Gama-Sosa et al., 1983; Schroeder et al., 2015; Tsien et al., 2002). Our work suggests that differences in global CpG methylation between trophoblasts and somatic cells are much smaller than previously reported. There are several potential reasons for these discrepancies. Firstly, our analysis clearly demonstrates that somatic cell populations have varying degrees of CpG methylation, and thus relative to some somatic tissue types (neutrophils, B-cells, hepatocytes), placental lysates and trophoblasts are indeed hypomethylated. Indeed, studies reporting global methylation in the placenta frequently use immune cell populations that can be easily harvested from the peripheral blood as comparators (Chatterjee et al., 2016; Ehrlich et al., 1982; Fuke et al., 2004; Schroeder et al., 2013). Secondly, four of the seven previous studies (Ehrlich et al., 1982; Fuke et al., 2004; Gama-Sosa et al., 1983; Tsien et al., 2002) investigating global placental DNA methylation employed high-performance liquid chromatography (HPLC). HPLC only allows quantification of the relative ratio of methylated cytosine residues against unmethylated cytosines and cannot localise individual CpG-site methylation changes, which is possible with the more sensitive RRBS technique (Kurdyukov and Bullock, 2016). However, RRBS does have limitations, such as having less sensitivity to methylation changes in regions with low CpG density. Future studies employing alternative techniques for quantifying methylation, such as MethylC-seq, which can distinguish between 5-methylcytosine (5mC) and 5-hydroxymethylcytosine (5hmC), would aid in allowing a more complete understanding of human trophoblast methylation (Urich et al., 2015).

### Conclusions

In conclusion, placental trophoblasts are uniquely methylated, but as there is only a small difference in global CpG methylation between trophoblasts and many somatic cells, it is likely that the distinct patterns of methylation in trophoblasts play a more important biological role than the overall extent of methylation. Furthermore, the small difference in global CpG methylation observed between trophoblasts and many somatic cell types may stem from blastocyst development where global CpG methylation does not appear to significantly decrease with trophectoderm differentiation in the human as it does in the mouse.

## MATERIALS AND METHODS

### Trophoblast isolation

Use of first trimester placental tissue (8.0–12.1 weeks of gestation) in this study was approved by the Northern Regional Ethics Committee (NTX/12/06/057/AM04). Hoechst-low side-population trophoblasts (
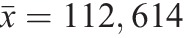
 cells/sample, *n*=4), Hoechst-high ß4-integrin positive cytotrophoblasts (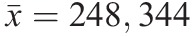 cells/sample, *n*=4) and HLA-G positive extravillous trophoblasts (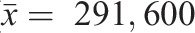 cells/sample, *n*=4) were isolated from the same four first trimester human placentae using fluorescence activated cell sorting as previously described (James et al., 2015). In brief, first trimester villous tissue underwent two enzymatic diests in 10 ml of phosphate buffered saline (PBS) containing 0.25% trypsin (Gibco) and 200 μg/ml DNAse I (Sigma-Aldrich). Cells from the first digest were stained with 5 μg/ml of FITC-conjugated anti-human HLA-G antibodies (AB7904, Abcam). Cells from the second digest were stained with 4.5  μg/ml of AlexaFluor647-conjugated anti-human HLA-A, B, C antibodies (Clone W6/32, #311414, BioLegend, San Diego, USA), and 10  μg/ml of FITC-conjugated anti-human ß4 integrin antibodies (AB22486, Abcam) for 30 min at 37°C before incubation with 10 μg/ml Hoechst 33342 for 90 min at 37°C (Sigma-Aldrich). To inhibit Hoechst 33342 efflux (negative control) an aliquot of unstained cells from the second digest was also incubated with 10 μg/ml Hoechst 33342, as above, in the presence of 10 μM fumitremorgin C (Sigma-Aldrich) and 150 µM reserpine (Sigma-Aldrich). To exclude dead cells, cells from both digests were stained with 1 μg/ml propidium iodide (Invitrogen).

Following staining, cells were sorted into 1.5 ml Eppendorf tubes containing 200 µl of PBS using an Aria II SORP (Becton Dickinson, Franklin Lakes, USA). To do this, non-viable cells labelled with propidium iodide (Fig. 2A) and doublets (data not shown) were excluded. For the first digest, HLA-G-FITC positive extravillous trophoblasts were gated relative to unstained controls (Fig. 2B). For the second digest, any HLA-A, B, C positive contaminating mesenchymal cells were excluded. The side-population gate was set based on the fumitremorgin-C and reserpine treated negative control, which contains no Hoescht 33342-low cells (Fig. 2C). Side-population trophoblasts were then sorted by capturing Hoescht 33342-low cells present in this gate in the main sample (Fig. 2D). A gate was drawn around the main Hoescht 33342-high population, and ß4 integrin-FITC positive cytotrophoblasts (relative to the negative control) were sorted from this population (Fig. 2E).

### RRBS

DNA from each of the three trophoblast populations was extracted using a Qiagen DNA Mini Kit. DNA quantity was measured using a Qubit^®^ dsDNA HS Kit (Molecular Probes, Eugene, USA) or Qubit^®^ dsDNA BR Kit (Molecular Probes). RRBS was performed on 500 ng of DNA per sample as a service by New Zealand Genomics Limited (NZGL, Dunedin, New Zealand) as previously described (Chatterjee et al., 2012a) using the *MspI* restriction enzyme in the RRBS library preparation with one size selection step (150–325 bp). Libraries were amplified with 15–18 cycles.

### Data clean up and analysis

RRBS libraries underwent single-ended (100 bp) sequencing using an Illumina HiSeq2000 (Chatterjee et al., 2012b). The reads were aligned to human GRCh37 reference genome assembly using Bismark aligner (Krueger and Andrews, 2011). The resulting bam files were sorted and Bismark methylation extractor (Krueger and Andrews, 2011) was used to determine DNA methylation status and to yield CpG report files. As RRBS enriches for CpG islands (which are usually unmethylated), methylation measurements by RRBS are expected to be lower than for the whole genome, but this was mitigated in our analysis by prior removal of CpG island specific data (Ficz et al., 2013; Peat et al., 2014). Finally, analysis of these report files was performed with ‘methylKit’ (Akalin et al., 2012), which assesses methylation at individual CpG sites [it counts of the number of methylated (T) versus unmethylated (C) bases at each CpG], performs differential methylation analysis (logistic regression with FDR to control for false positives), determines average global methylation levels for each cell type, identifies which genomic elements methylation events occur, and performs a principal components analysis (PCA). Significantly methylated regions were those with a q-value of less than 0.01 and methylation difference exceeding 25%. The mean global CpG methylation across the sample was calculated and reported with the standard error of the mean (S.E.M.). The total number of methylated CpG sites was identified for each sample and the proportion of methylated CpG in exon, intron, promoter and intergenic regions established then averaged for each cell/tissue type. The percentage of CpG sites overlapping with genomic elements was determined with promoter>exon>intron precedence. Data were analysed statistically by one-way ANOVA followed by a Bonferroni post-test, or by Student's *t*-test for two group comparisons, using GraphPad PRISM (v7, GraphPad). Percentage methylation distribution and coverage per base information are provided in Table 1. These data have been deposited in the Gene Expression Omnibus (GEO) database with accession number GSE109682.

### Acquisition of publicly available data

Publicly available raw data (Table 1) was identified using NCBI GEO and downloaded using Aspera Connect. The SRA tool kit was used to obtain .fastq files which were processed as described above.
